# Measuring value sensitivity in medicine

**DOI:** 10.1186/s12910-016-0164-7

**Published:** 2017-01-28

**Authors:** Christian Ineichen, Markus Christen, Carmen Tanner

**Affiliations:** 1 Institute of Biomedical Ethics and History of Medicine, University of Zurich, Winterthurerstrasse 30, 8006 Zurich, Switzerland; 2 University Research Priority Program Ethics, Zollikerstrasse 117, 8008 Zurich, Switzerland; 3 Leadership Excellence Institute Zeppelin, Zeppelin University, Am Seemooser Horn 20, 88045 Friedrichshafen, Germany; 4 Department of Banking and Finance, University of Zurich, Plattenstrasse 32, 8032 Zurich, Switzerland

**Keywords:** Value sensitivity, Moral sensitivity, Ethical sensitivity, Moral values, Moral competences, Medical ethics training

## Abstract

**Background:**

Value sensitivity – the ability to recognize value-related issues when they arise in practice – is an indispensable competence for medical practitioners to enter decision-making processes related to ethical questions. However, the psychological competence of value sensitivity is seldom an explicit subject in the training of medical professionals. In this contribution, we outline the traditional concept of moral sensitivity in medicine and its revised form conceptualized as value sensitivity and we propose an instrument that measures value sensitivity.

**Methods:**

We developed an instrument for assessing the sensitivity for three value groups (moral-related values, values related to the principles of biomedical ethics, strategy-related values) in a four step procedure: 1) value identification (*n =* 317); 2) value representation (*n =* 317); 3) vignette construction and quality evaluation (*n =* 37); and 4) instrument validation by comparing nursing professionals with hospital managers (*n =* 48).

**Results:**

We find that nursing professionals recognize and ascribe importance to principle-related issues more than professionals from hospital management. The latter are more likely to recognize and ascribe importance to strategy-related issues.

**Conclusions:**

These hypothesis-driven results demonstrate the discriminatory power of our newly developed instrument, which makes it useful not only for health care professionals in practice but for students and people working in the clinical context as well.

**Electronic supplementary material:**

The online version of this article (doi:10.1186/s12910-016-0164-7) contains supplementary material, which is available to authorized users.

## Background

In medicine, there is a need to emphasize the psychological prerequisites for clinical acting [1]. Generally, psychological moral competencies [2, 3] of medical professionals are rarely assessed, and there are hardly any instruments that are appropriate to measure such competencies. One such relevant competence is value sensitivity, a prerequisite for moral decision-making and behavior. Based on previous research (identification and characterization of domain specific values [4]), we aimed at developing an instrument which measures value sensitivity in medicine. The instrument aims at supporting medical professionals and their patients by empowering healthcare practitioners to recognize issues with ethical relevance when they arise in practice. With this work, we outline the procedural steps necessary for the development of the instrument. Finally, we describe the validation of the instrument by means of a group comparison.

### The concept of moral sensitivity

The discussion regarding sensitivity for values has traditionally focused on moral values, broadly construed as standards of what is valuable or important in certain issues or situations. Moral sensitivity (also referred to as moral awareness or ethical sensitivity/sensibility) then is traditionally defined as the ability to recognize moral issues when they arise in practice [3, 5–8], see also [9] or [10] for reviews on varying definitions of the construct]. More precisely, moral sensitivity incorporates both the ability to recognize moral issues in a morally ambiguous situation and the ascription of importance to these same issues [11]. It includes being responsive to the needs of others in addition to anticipating whether a course of action can harm or help others or whether it violates internalized moral standards or codes of conduct that are usually concretions of values. In line with this conceptualization, we adopt the definition of moral sensitivity that includes both the recognition and the ascription of importance to moral values. Accordingly, moral sensitivity is proposed to cover both, an intuitive- (quick, reflexive recognition of a morally relevant aspect in a situation) and a deliberative process (vectored attentiveness to morally relevant aspects).

Lack of moral sensitivity — also called moral blindness — is likely to have far-reaching consequences. Researchers found that “morally blind” people can act with the best of intentions but behave in contradiction to their own values and principles, without being aware of it [12]. If the moral issues at stake are not identified, no moral problem will exist for the individual and therefore there will be no need to enter into a moral problem-solving phase [13]. Thus, it is obvious that without a certain moral awareness, there is no reason to question one’s or other’s behaviors from a moral point of view. Consequently, without moral sensitivity, professionals may not be able to appropriately recognize, interpret and respond to the concerns of patients and their relatives.

Although many researchers agree on categorizing moral sensitivity as a prerequisite for the initiation of moral decision making (e.g. [6, 13]), past research has focused more on the development of instruments for measuring the latter while largely neglecting the former [14]. The Dental Ethical Sensitivity Test (DEST) by Bebeau and Rest (1982) [15] is the oldest measure of moral sensitivity and was created to measure individuals’ ability to identify ethical issues and deviations from professional codes of ethics in dental practice. More recent attempts focus on the measurement of moral sensitivity in the business domain (e.g., in accounting or business situations). In 2007, Jordan [9] provided a comprehensive review and critical evaluation of the available measurements, pointing out that there is still a great need for validated measures of moral sensitivity (see also [16] for an emphasis of the medical context). A concise evaluation of those tests indicates that current instruments fall short regarding several aspects (see also [9]). For example, they often lack an evaluation based on criteria of diagnostic test theory. Our research project aims at developing an instrument for the measure of moral sensitivity which overcomes such pitfalls and is part of a comprehensive theory of moral intelligence [3].

We will expand the concept of moral sensitivity by considering a broader spectrum of values. The reason for this is that the current research on moral sensitivity focuses on values whose relation to morality is undisputed both from a theoretical perspective (i.e., they are discussed as prototypical moral values in the ethics literature) and based on empirical findings (i.e., people consider those values to be *moral* values). Examples include benevolence, honesty, or fairness. However, in professional contexts, other values may be relevant as well, although they may not be perceived as moral values (e.g. cost-effectiveness or reputation). By the term ‘value’ we refer to stable beliefs about desirable states or conducts of behavior, which serve as general normative standards to judge and justify actions not necessarily related to ethics [17]. Therefore, we suggest that an assessment of moral sensitivity should include values that are not intuitively perceived as moral but that refer to legitimate claims within the specified domain. By “domain”, we refer to any social sector, for example, professional fields such as medicine or business, associated with a specific set of values that are considered to be important in that sector. In the following, we therefore refer to the more general notion of “value sensitivity”. Based on previous research [4], value sensitivity in the research context of medicine is composed of three subcomponents: sensitivity for moral-related, principle-related and strategy-related values.

### The relevance of value sensitivity in medicine

The medical domain exceptionally challenges ones’ moral competencies because of numerous problems in that domain. These include actions under time pressure, inclusion of high-level moral values (e.g. non-maleficence) and dilemmas involving numerous stakeholders apart from structural barriers. Much of the controversial discussion focuses on codes of conduct. Some authors certify that these codes have only minor impacts on daily practice [18] and found that nurses evaluate them as being of little use [19]. Some studies investigating the success of teaching medical ethics even observed that the student’s moral sensitivity diminished over the course [20]. This result may indicate that traditional teaching strategies tend to overlook the key competence of recognizing moral aspects in ambiguous clinical situations. In the past, authors such as Kleinmann complained about the neglect to promote psychological competences in teaching programs [1]. Consequently, the question of which specific psychological abilities have to be trained to realize such competency has not been adequately emphasized. Accordingly, we point out that moral behavior is not solely reflected in knowledge about ethics but also by paying attention to ones’ psychological competencies. Both aspects should be included in medical education.

If moral behavior rests on moral competencies, the need for tools to measure the baseline status with the possibility of training such skills becomes an important undertaking [21]. We suggest that one way of supporting health care professionals’ training is to allow them to learn about their individual strengths and weaknesses with respect to their own value sensitivity. In medicine, it is important to be able to obtain a swift recognition of which values are involved in a particular situation and which stakeholders could be affected by the ethical decision (e.g. patient, physician, or close relatives). Additively, the body of evidence suggests that people differ quite substantially in terms of moral- (or more generally value-) sensitivity [9, 11, 22–29]. Therefore, we consider it as an imperative to holistically integrate the insights of recent moral (psychological) research about the conditions of human moral ability into medicine in general and in the process of education of medical professionals in particular. Potential applications of our model are: (1) as a diagnostic tool for medical professionals in order to mirror possible strengths and weaknesses, (2) as an educational tool in the context of medical school and (3) as an instrument for advanced training of individuals, who work, for example, as clinical ethicists.

## Methods

### Developing an instrument for measuring value sensitivity

As outlined in the last paragraph of section “The concept of moral sensitivity”, we embed moral sensitivity into the broader concept of value sensitivity. We performed steps towards developing an instrument designed to assess sensitivity towards three value groups. First, based on previous research we gained empirical evidence for a domain-specific value selection as well as insights into what extent the values are perceived as moral values (step 1: value identification). Notably, domain-specificity may include the possibility that the perceived morality of values differ between domains. Thus, in business compared to medicine, it is likely that other values are deemed important and shared values might cluster differently. This relates to the work of Bebeau and Thoma [30] who used the term “intermediate ethical constructs” to refer to profession-specific concepts within a given domain (e.g. in medicine: professional autonomy, informed consent, privacy). Second, we obtained representative statements which were used as stimulus material instead of naming the values explicitly (step 2: value representation). Third, we developed and validated morally ambiguous vignettes characteristic of the clinical context (step 3: vignette construction and selection). These three steps are described below. In paragraph “Testing the validity of the value-sensitivity measure”, we describe the validation of the instrument by means of a group comparison.

**Fig. 1:**
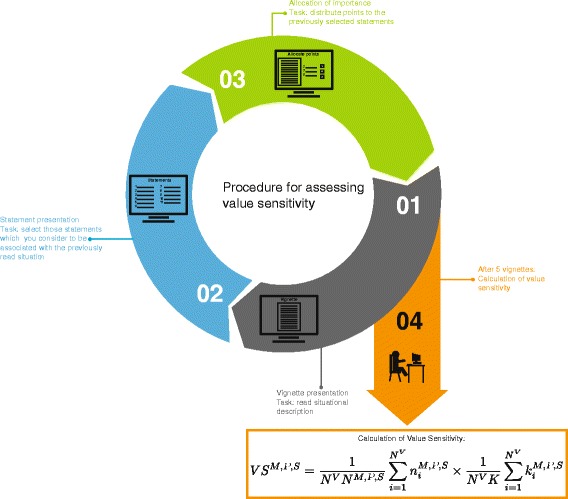
A situational description (vignette) is presented on the computer screen: after having read one vignette which disappears upon clicking the “next”-button, participants are provided with a list of value-related statements. They are asked to select those statements which they consider to be associated with the previously read situation. This task is designed to examine which values participants recognize in each vignette. Next, the vignette reappears together with all previously chosen statements and participants are asked to distribute points (i.e. allocate importance) to these same statements. This task is designed to assess the perceived importance of the selected value. Note: The formula denotes how value sensitivity is currently calculated: the mean of recognized values for each cluster (e.g. strategy-related values) is multiplied with the normalized number of points allocated to the corresponding value-cluster

The functioning of the instrument is summarized as follows (see Fig. 1 below): Using a vignette-based approach, the instrument has been designed to present people with morally ambiguous situational descriptions and to investigate 1) which values they are more or less likely to identify and 2) which values they consider important within the presented scenario. After vignette presentation, respondents were provided with a list of value-related statements (items, see step 2: value representation) among which they could choose. The extent to which respondents to several vignettes choose more moral-related values and rank them as more important relative to other categories of values is indicative for moral-related value sensitivity.

All studies in this research project were conducted in accordance with the ethical review processes of the University of Zurich and the checklist for the ethical evaluation of empirical studies that don’t need mandatory authorization (CEBES) of the Institute of Biomedical Ethics and History of Medicine (http://www.ibme.uzh.ch/en/biomedicalethics/forschung/cebesEN.html) and were analyzed using the software package SPSS Version 23.

### Step 1: value identification

A general challenge when assessing value sensitivity is the identification of relevant values for the domain under consideration. There is also the challenge of investigating to what extent these values are perceived as moral— or non-moral in order to develop balanced vignettes that include several different values and that reflect the moral ambiguity of a particular situation (see step 3).

#### Participants and Procedures

In order to examine which values are perceived as being examples of moral or other categories of values, a sample of medical students and professionals (*n =* 317; 54.3% females, mean age: 26.6) was asked to evaluate a number of values along four moral-related dimensions (for more details see [4]).

#### Outcome

The main outcome involved an empirically informed classification of distinct value-clusters along the moral vs. strategic (non-moral) continuum. Based on that, we were able to classify 11 values in three clusters: A first cluster (the “general morality-related cluster”) was composed of the values of responsibility, honesty, loyalty and respect. These values obtained higher ratings on all moral-related dimensions, suggesting that they were perceived as examples of moral values. A second cluster (“strategy-related cluster”) included the values performance, cost-efficiency and reputation. These values received consistently low ratings on all dimensions, suggesting that they were perceived as unrelated to moral aspects. Interestingly, we found that all values associated with the principles of biomedical ethics [31] – non-maleficence, justice, beneficence (in our context: care, for an explanation see [4]) and autonomy — formed a separate, third cluster (“principle-related cluster”). Based on the ratings, those values were between the other two clusters, yet closer to the moral than to the strategy-related value-cluster. Due to their importance in training medical personnel in biomedical ethics, this group was included in the instrument development as well. (For an in-depth description of the rationale for selecting values and their categorization into three groups, see [4]). Notably, some of the values (care, non-maleficence, loyalty, justice) relate to Haidt’s moral foundations [32], although not all of them received ratings such that they have been classified in the general morality-related cluster.

### Step 2: value representation

Since our assessment of individuals’ sensitivity to particular categories of values is based on the kind of value-related items that people consider relevant for the situation contained in the vignette, another challenge was to minimize the flaw of provoking socially desirable answers. Such a risk may be likely when values (such as honesty, fairness, and the like) are explicitly named in an item. A central issue was therefore to avoid naming the value in the wording of the items, such that the presentation of stimulus material to subjects did not guide participants to attend to ethical issues. The method used here was designed to minimize this flaw. Furthermore, providing only a single value term involves the risk that the meaning of the term is under-determined for the participant. In step 2 of the construction phase, we therefore wanted to obtain adequate descriptions of such values, preferably by using short statements for each value describing typical behavioral manifestations of the corresponding value. The statements had to fulfill the criteria (1) of inherent and related normativity (i.e. they had to be similar to the values they describe), (2) they should incorporate the perspectives of different stakeholders (patients, doctors; hospitals and care-centers), (3) they should incorporate different behavioral actions which match the values, and (4) finally they should be synonymous, as opposed to explicitly naming the value at stake. In order to present material for quality-assessment, we developed four statements for each value.

#### Participants and Procedures

The statements were presented in randomized order. Participants (same sample as in step 1, *n =* 317) quality-checked each statement using a bipolar 6-point Likert scale ranging from 1 (not representative at all) to 6 (very representative). In order to effectively avoid socially desirable answer tendencies, we included one distractor-statement in every set of statements. For example, one distractor-statement describing “performance” was included in a set of four statements describing “autonomy”. Accordingly, participants were presented with five statements per value. As distractors, we used preliminary developed but superfluous value statements.

#### Outcome

In order to obtain representative statements for each value, we calculated the mean of each statement based on the Likert scale evaluation of participants (due to the vast number of statements, the whole list of statements is presented in Table 1a, b in Additional file 1 only). Statements resembled the following structure: For care we used “A physician or a caregiver should provide assistance to patients who cannot help themselves”. A high mean value indicated high representativeness. Statements were selected only if their mean value was above 4.5. Six out of 44 statements did not meet this criterion and were excluded for step 3 and the validation study. After representativeness-testing, we retrieved two to three statements for each value which could be integrated as stimulus material into our instrument.

Most distractors achieved the lowest mean-values for each value-group (9 of 11; see Additional file 1: Table S2 Distractor analysis). In one case including the value “responsibility” where the distractor achieved a higher mean value, the distractor was inappropriately chosen due to excessive semantic overlap (distractor for responsibility was “care”). We also conducted t-tests for verifying statistical significant differences between distractors and the mean-wise lowest statement for every value (analysis performed prior to exclusion of any statement). Results demonstrated a statistically significant effect on 8 of the 11 comparisons (see Additional file 1: Table S2 Distractor analysis).

### Step 3: vignette construction and selection

Step 3 consisted of constructing vignettes that describe ambiguous conflict situations that do not pull extensively in a specific value-direction (verified by analyzing the more or less even distribution of values selected by participants) and that are relevant for the medical context. Based on a literature research and interviews with medical experts in Switzerland, we developed 12 vignettes of approximately equal length, morally ambiguous content and the integration of multiple stakeholders. Prior to study inclusion for quality assessment, these vignettes were reviewed by external experts from medical ethics.

#### Participants and procedures

After assessing demographic information (gender, age, field of study, number of completed semesters) and information about participants’ work experience in medicine (whether they have work experience, and what kind of experience), participants (*n =* 37; 78.4% females, mean age M = 25.9 years, 62% medical students, 16% from nursing school, 45.9% reported to have work experience) were instructed to put themselves in the role of a clinical expert in charge who is partly involved as a committee member of a clinical expert-group. They were told that currently, there were six cases (2 cohorts, 6 cases/vignettes per cohort) to be discussed during the next committee meeting. In this way, the participants were primed in a similar way as in the main study (see validation study, below). Then, the participants were asked to evaluate the vignettes according to the following quality criteria: (1) comprehensibility, (2) required level of expert-knowledge, (3) relation to reality, (4) extent of achievement-oriented, reputation-related or economic-related content and (5) extent of moral-related or social-oriented content using a bipolar 5-point-Likert scale. (4) and (5) were assessed as quality criteria for moral ambiguity (i.e. balanced involvement of moral and strategic aspects in the vignette, [6]). Moral ambiguity is a vital prerequisite of vignettes to prevent biased responses. The vignettes were all between 137 and 202 words long, developed and preselected by two writers and one external reviewer and written based on the results of steps 1 and 2.

#### Outcome

We calculated the means of the 5-point-Likert scale evaluations regarding quality and moral ambiguity of the vignettes (the results of the descriptive analysis are given in Table 1). Vignettes were selected for further review if (1) comprehensibility was achieved (i.e. mean values ≤ 2.5), if (2) the requirement of expert-knowledge was moderate (i.e. mean values between 1.5 and 3.5), if (3) relation to reality was high (i.e. mean values < 2.5) and if (4) the vignettes incorporated both strategic as well as moral aspects but not to a very obvious but rather ambiguous extent (i.e. mean values < 4.5).

**Table 1:** Mean value quality analysis of vignettes

Quality-criteria							Moral ambiguity					
	Comprehensibility		Expert knowledge		Relation to reality		Strategic		Moral		Δ	
Vignette No	M	(SD)	M	(SD)	M	(SD)	M	(SD)	M	(SD)	(SD)	*p*
**1**	1.63	(0.89)	1.69	(0.80)	1.62	(0.62)	4.19	(0.98)	4.19	(0.75)	(1.42)	1.000
2	1.76	(0.83)	1.94	(1.03)	2.12	(0.86)	4.65	(0.79)	3.41	(1.00)	(1.25)	0.001**
**3**	1.63	(0.89)	2.81	(1.11)	1.38	(0.62)	3.38	(1.54)	3.88	(0.89)	(1.86)	0.300
4	1.82	(0.81)	2.59	(1.00)	2.18	(0.88)	4.59	(0.62)	3.82	(1.02)	(1.26)	0.023*
**5**	1.41	(0.87)	1.59	(1.00)	2.24	(0.75)	4.18	(0.95)	4.29	(0.85)	(0.78)	0.543
**6**	2.5	(1.32)	2.63	(1.31)	1.69	(0.70)	3.53	(1.06)	3.53	(0.83)	(1.46)	1.000
**7**	1.89	(0.94)	3.16	(1.07)	1.79	(0.86)	4.21	(1.03)	4.26	(0.93)	(1.13)	0.841
8	1.35	(0.59)	2.35	(1.23)	1.75	(0.91)	4.1	(0.97)	4.95	(0.22)	(0.99)	0.001**
9	1.84	(1.17)	3.26	(1.05)	2.47	(1.17)	2.53	(1.07)	4.21	(0.98)	(1.29)	0.000***
10	1.52	(0.81)	2.62	(1.12)	1.29	(0.56)	3.81	(1.03)	4.9	(0.30)	(1.04)	0.000***
11	1.83	(0.86)	2.33	(1.24)	2.5	(0.86)	4.61	(1.15)	3.56	(1.34)	(1.83)	0.026*
12	1.62	(0.5)	2.75	(0.93)	1.75	(0.68)	3.44	(1.41)	4.75	(0.78)	(1.49)	0.003**

remark : vignettes (**bold**) were selected if: Mcomprehensibility ≤ 2.5, 1.5 < Mexpert_knowledge < 3.5, Mrelation_to_reality < 2.5, Mmoral/strategic < 4.5, Δns; *p < .05, ** *p <* .01, *** *p* < .001

A pivotal criterion was the balance between strategic and moral elements in each vignette (i.e. moral ambiguity). To test moral ambiguity and based on the average rating (M_Moral_, M_Strategic_), we conducted single t-Tests to test for dissimilarity. If means differed significantly, the vignette was excluded. Moreover, a Shapiro-Wilk test was conducted and yielded a significant result highlighting non-normality of the data due to the low sample sizes of the preselected vignettes. Therefore, a non-parametric Wilcoxon signed-rank test was executed. All results of the single t-Tests were confirmed. Five vignettes (number 1, 3, 5, 6 and 7, see Table 1) fulfilled the necessary criteria and were included as stimulus material in our instrument. The five chosen vignettes involved work-place problems within a clinic (V1 & V3), conflict within a nursing home (V5) and two vignettes including research issues in neurology (V6 & V7). The results are described for the selected vignettes only (the selected vignettes are displayed in Additional file 1: Selected Vignettes).

As mentioned earlier, the previously described three steps encompassed the first phase in the development of our value-sensitivity measure. In what follows, we advance this work by providing a first validity test of this measure.

## Results

### Testing the validity of the value-sensitivity measure

#### Hypothesis generation for expected group differences

In our final step, we aimed at demonstrating the validity of the measure by making use of a group comparison.

Hypothesis generation for the group comparison included some theoretical concepts: Regarding the reasons for inter-individual differences in perceiving moral issues in ambiguous situations, contemporary research predominately refers to social cognition theory (e.g., [33]) positing that individuals hold cognitive schemas (i.e. cognitive representations) depending on socialization. These models also imply that priming (activating a concept by providing external stimuli such as a word, a picture or an object; [34]) of a representation would foster its future activation by increasing its accessibility. Consistent with this, a substantial body of research clearly demonstrated that (consciously or subconsciously) primed information guide attention, encoding and the categorization of the situation by making concepts temporarily more accessible (for an overview see [35–37]).

Some schemas are chronically accessible in that they become automatically and habitually activated [36, 38]. Examples of chronic accessible representations are strong attitudes and deeply held values, beliefs or traits that are central to one’s identity or culture. In line with this, moral standards or values are acknowledged as moral schemas that vary in their accessibility [2, 11, 39]. Hence, individuals whose moral schemas are more accessible or even chronically accessible are expected to be more likely to direct attention more or less automatically and swiftly to moral issues — the same holds for schemas that represent other types of issues (e.g., strategic issues). For example, Jordan [11] has argued that business managers are less likely to detect moral-related dimensions than academics, because business managers have business rather than moral schemas guiding their attention and information processing more dominantly. Overall, researchers in moral psychology consistently conceive the activation and accessibility of moral schemas as crucial conditions of demonstrating moral sensitivity [9, 23, 40–42].

Based on the above delineation of the underlying theoretical concepts, our working hypothesis is that our instrument demonstrates ample discriminatory power between two groups of participants: care-professionals and professionals from hospital management. This hypothesis is built according to social cognition theory proposing that socialization in various working contexts shapes people’s cognitive schema (e.g. [11]). It is well-known from previous research that schemas strongly influence information processing, making people more likely to attend to, encode and recall information which match with the existing schemas (e.g., [36, 43]). In line with this, and because of being embedded in a working environment that expects from its members an orientation towards the principles of biomedical ethics which are also part of nurses’ training programs in ethics, we hypothesized nurses to be more likely to demonstrate greater sensitivity for principle-related issues. Of note is the fact that although the principles form a separate cluster, this cluster has a much stronger affinity to the moral as opposed to the strategic cluster. In contrast, we hypothesized that members of hospital management demonstrate greater sensitivity for strategy-related issues. This is because they are more often faced with strategy— and business-related problems in their working life. We were indifferent about the expectations related to the other, more general moral values. Since both groups may be faced with problems tapping into issues of e.g. honesty or fairness, both may have evolved some sensitivity for such issues. Of main interest is, whether our measure is capable of revealing the expected group differences, supporting the validity of our instrument.

In conducting the group comparison, we recruited professionals from nursing on the one hand and from hospital management, administration and human resources on the other. As outlined, we aimed at demonstrating the instrument’s capability to differentiate between these two cohorts (professionals from management: increased sensitivity for strategy-related issues, nursing professionals: increased sensitivity for values relevant in clinical practice, i.e. principle-related values).

#### Participants and procedures

In this study, 57 participants from various clinics located in the German part of Switzerland filled out the questionnaire. After rigorous examination of the data, 48 datasets fulfilled our quality criteria: participants were required to self-categorize them to one of the two groups and were required to have patient contact either on a daily basis (nursing professionals) or fewer than once a month (hospital management). 37 (30 females) were nursing professionals whereas 11 (7 males) worked in the field of hospital management, human resources or administration. There was a statistically significant gender misbalance and mean age difference (mean age: nursing: 39 years, management: 48 years). This gender misbalance, however, is not surprising given that nursing and management are among the most sharply sex-segregated of occupations. The two groups did not significantly differ in the time needed for completing the questionnaire (see Table 2).

**Table 2:** Demographic differences of groups

	Nursing	Management	Significance level
Number of participants	37	11	
Mean age [years]	39	48	MW: *p =* 0.01
Years of employment	17	15	n.s.
Gender-ratio	f: 30, m: 7	f: 4, m: 7	MW: *p =* 0.005
Time needed for filling out questionnaire [min]	39	44	n.s.

In the questionnaire, we first assessed demographic information (gender, age, field of work) and information about participant’s work experience in medicine (duration and frequency of contact to patients). Participants then were briefly instructed how to fill out the questionnaire. Subsequently, they were asked to put themselves into the role of a clinical expert in charge who is partly involved as a committee member of a clinical expert-group. They were told that currently, there were five cases (i.e. vignettes) to be discussed during the upcoming committee meeting during which they were expected to bring in spontaneously the considerations they deem important for the case evaluation. We explicitly noted that decisions about which concrete actions to take would be elaborated at a later time. In order to prevent biased responses, we also refrained from telling respondents that there are moral issues in the presented vignettes.

Next, the five vignettes were randomly presented on the computer screen: after having read one vignette which disappeared upon clicking the “next”-button, participants were provided with a list of 11 value-related statements (see step 2: value representation). Thus, participants were provided with all 11 values from the three clusters (i.e. in form of 11 statements) for the value selection step following vignette inspection. For most values, there were multiple statements which satisfied quality criteria (see step 2: value representation). Hence, we presented participants with the most suitable statements to content. They were asked to select those statements which they consider to be associated with the situation. They could select as many statements as they liked. This task was designed to examine which values participants recognize in each vignette. Next, the vignette reappeared together with all previously chosen statements, and participants were asked to distribute points (i.e. allocate importance) to these statements. In total, 10 points had to be distributed to the statements, including the possibility that some statements could receive 0 points. This task was designed to assess the perceived importance of the selected value (see Fig. 1 for instrument process illustration purposes).

This procedure negates the possibility of “wrong” answers, e.g. by choosing statements that reflect values where one may argue that they are not directly involved in the vignette. Since reality is often ambiguous it is up to individuals to interpret the situation. We know that human perception is highly subjective and selective, but dominant schemas do affect the perception process. For example, morally sensitive individuals are generally more likely to associate moral values within ambiguous situations because they are expected to have more easily accessible moral schemas. As a result, they are also expected to be more likely to direct attention more or less automatically and swiftly to moral issues (see “Hypothesis generation for expected group differences”). The same holds for individuals that show a higher sensitivity for strategic or principle-related values. This aligns with previous conceptualizations (e.g. see [42]) after which and as opposed to other ethics constructs (e.g. formalism) which hold a clear position on what is right and wrong, value sensitivity has additional explanatory meaningfulness in simply acknowledging that individuals are considering concepts associated with morality. Additionally, categorizing a perceived value as wrong in itself is a moral claim which would need justification in such a way that it would introduce the challenge of defining a deciding authority (i.e. who decides about the wrongness of a given value). In our view, the process of perception of values is a hermeneutical rather than absolutist process. Overall, we posit that flexible and pluralistic value-perception, theoretically speaking, is positive even if this complicates moral decision-making and behavior at a later stage. Specifically, we hypothesize that multiple value perception reduces the risk of moral blindness [44] and hence unethical behavior. Multiplicity in value recognition, however, should not be understood as a form of relativism. Our notion refers to other claims [44] that good decisions depend on perspective-taking and imagination and thus the ability of an individual to appraise multiple aspects during decision making. It also supports tolerance rather than fundamentalism.

#### Calculating value sensitivity

Participant’s value sensitivity scores were calculated by combining both a) the number of values recognized in each vignette and b) the importance attributed to these recognized values operationalized as the number of points assigned to those selected values. In this respect, value sensitivity is congruent with previous definitions of moral sensitivity as containing both the recognition and the ascription of importance of moral-related issues (see [6, 11, 42]). In order to calculate participant’s value sensitivity for the three clusters, the mean number of recognized values for each cluster separately of the aggregated data of all five vignettes was calculated. Next, we calculated the total number of allocated points for each cluster and divided it by the total number of possible points. The mean of recognized values for each cluster was then multiplied with the normalized number of points allocated to the corresponding value-cluster.^1^ Hence, we summed all recognized principle-related values across all vignettes and calculated the mean which was multiplied by the normalized value of points allocated to principle-related values for all vignettes in order to calculate the sensitivity for principle-related values. We calculated the sensitivity for the other two value clusters in the same way. Consequently, potential differences in perception between vignettes carried less weight. By introducing a forced format with regard to the distribution of points of importance, dependency of the three constructs emerges. Because we align the conceptualization of moral sensitivity to our construct, we deliberately included the explicit task of prioritization of previously recognized issues (see [11]).

Formally, the computation of value sensitivity is as follows: Let *VS* ^*M*^, *VS* ^*P*^, and *VS* ^*S*^ denote the value sensitivity for moral-related values (*M*), principle-related values (*P*) or strategy-related values (*S*). Furthermore, let *N* ^*V*^ be the number of vignettes used; *K* be the number of points that can be distributed to all chosen values per vignette; and *N* ^*M*^, *N* ^*P*^ and *N* ^*S*^ be the number of values per value group (*M*, *P* or *S*). Finally, let *n*_*i*_^*M*^, *n*_*i*_^*P*^and *n*_*i*_^*S*^ be the number of values chosen per vignette *i* and group; and *k*_*i*_^*M*^, *k*_*i*_^*P*^and *k*_*i*_^*S*^ be the number of points attributed to moral-related, principle-related and strategy-related values per vignette *i*, whereas *k*_*i*_^*M*^ + *k*_*i*_^*P*^ + *k*_*i*_^*S*^ = *K*. Then the generalized definition of value sensitivity is given as:

$$ V{S}^{M, P, S} = \frac{1}{N^V\ {N}^{M, P, S}}{\displaystyle \sum_{i=1}^{N^V}}{n}_i^{M, P, S} \times \frac{1}{N^V\  K}\ {\displaystyle \sum_{i=1}^{N^V}}{k}_i^{M, P, S} $$

In our case, *N* ^*V*^ *=* 5, *K = 10* and (for example) *N* ^M^ = 4. Thus, the sensitivity for generally moral values is calculated as:

$$ V{S}^M = \frac{1}{20}{\displaystyle \sum_{i=1}^5}{n}_i^M \times \frac{1}{50}\ {\displaystyle \sum_{i=1}^5}{k}_i^M $$

The calculation of *VS* ^*P*^ and *VS* ^*S*^ is analogous (*N* ^*P*^ = 4, *N* ^*S*^ = 3).

#### Outcome

A Shapiro-Wilk-test analyzing normality was performed and highlighted non-normality for sensitivity for strategy-related values. We therefore conducted a Mann–Whitney U Test as a non-parametric test in order to investigate group differences together with logistic regression. Statistical significance was accepted at a *p ≤ 0.05* level.

**Fig. 2:**
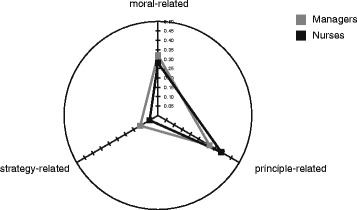
Group comparison for moral-related, principle-related and strategy-related value sensitivity

We compared value sensitivity of nurses and hospital managers for the three value groups (moral-related values, principle-related values, strategy-related values) (see Fig. 2). In terms of sensitivity for principle-related values, the group comparison yielded a significant difference with nurses (*M = 0.39*, *SD = 0.12*) revealing higher scores than management professionals (*M = 0.32, SD = 0.09*) (*U (125.00) = −1.93, p = 0.05*). In terms of sensitivity for strategy-related values, we found another significant difference between the groups, with professionals from hospital management revealing higher scores (*M = 0.11, SD = 0.08*) than nurses (*M = 0.05, SD = 0.07*) (*U (110.00) = −2.30, p = 0.02*). In terms of sensitivity for general moral-related values, we found no significant differences between the groups (*p > 0.35*). Consequently, our instrument was able to discriminate between nursing- and management professionals as our hypothesis predicted. Using logistic regression for investigating a main effect of group yielded a significant result (main effect: χ2(3) = 9.703, *p =* .021). Analysis of the main effect and visually examining the spider graph reveals a shift of the managers’ sensitivities relating to the strategic- and general moral value-cluster. It also reveals dependency of the 3 constructs based on the allocation of points of importance in step 2: because we deliberately included the conceptualization of prioritizing previously recognized issues (see [11]), denoting to deciding which values are to a greater degree important and which are not, the triangular shape representing normalized sensitivities to the three clusters is different in degree and not in kind.

Next, we tested for potential gender differences. A Mann–Whitney U test yielded the following results: males (*M = 0.11, SD = 0.09*) demonstrated a significantly greater sensitivity for strategy-related values compared to females (*M = 0.04, SD = 0.06*) (*U (122.50) = −2.63, p = 0.01*). On the other hand, females *(M = 0.39, SD = 0.12*) demonstrated a greater sensitivity for principle-related values compared to males (*M = 0.33, SD = 0.10*) *(U (152.50) = −1.94, p = 0.05*). There was no significant gender difference regarding sensitivity for generally moral-related values (*p > 0.65*).

Finally, the analysis of the frequency of each value selected by participants across vignettes demonstrated only minimal differences (6 comparisons of 110 involving 3 values (cost-efficiency, autonomy, loyalty — one of every cluster) yielded a significant result only). This may corroborate our negation of “wrong” value attribution (see Additional file 1: Table S3 for a table indicating the choices and what was selected by each group). Moreover, testing Cronbach’s alpha to examine the reliability of the overall scale provided a mixed result: although the recognition task provided a good result (general moral-related values: α = 0.736, strategy-related values: α = 0.836, principle-related values: α = 0.769), the overall scale yielded a highly satisfactory result only for the strategic dimension (general moral-related values: α = 0.514, strategy-related values: α = 0.742, principle-related values: α = 0.466) — which is, however, the dimension where the most robust group difference has been found. This indicates that the differentiation between the generally moral- and principle-related dimension might be reconsidered when improving the scale in future research.

## Discussion

In the context of this research project, we were successful in executing a first trial of validity testing of our developed tool for the measure of domain specific value-sensitivity. Significant differences emerged when comparing professionals from nursing compared with experts from hospital management and -administration. More specifically, nursing professionals demonstrated greater sensitivity for principle-related values, while professionals from hospital management and administration revealed greater sensitivity for strategy-related values. This confirms our hypothesis. Finally, groups did not significantly differ on more general moral-related values. As outlined previously, we think that both groups may be faced with problems tapping into issues such as honesty and fairness, which might explain the indifference of the two groups.

This study incorporates the following limitations. First, we are well aware of the fact that group comparisons are only one step of validating instruments. Future studies are needed to further test the instrument. In line with this, we are currently running studies aiming at assessing concurrent as well as convergent validity by comparing our instrument with other congruent or non-congruent questionnaires. Furthermore, we are in the process of complementing the instrument with an implicit measure. Hence, more research is needed for the development of an instrument that is able to assess value sensitivity at an individual level.

Second, due to the small sample size, hospital managers and administrators used in this study might not be representatives of their profession. Moreover, the generally low level of strategic sensitivity for both groups might have been extrinsically induced by application of an unbalanced stimulus material: both, the moral— and principle-related value clusters are composed of 4 values each, whereas the strategy-related value cluster only consisted of 3 values. Hence, the pure likelihood to choose a strategic value was smaller.

Third, one has to take into account that, based on the small sample size of at least one group and because of a non-randomized study design, there might be intrinsic differences between the individuals of the two groups related to gender. Furthermore, we are unable to fully explore the effects of gender detached from the domain of occupation, since gender was endogenous to domain of occupation. The influence of gender on morality is controversially discussed and appears also to depend on age, level of education, occupation and their interrelation [45]. Whilst studies investigating gender differences are largely controversial, in most cases when gender differences can be identified, women tend to show greater levels of moral awareness, particularly when studies focus more closely on care-based moral convictions [29, 46].

Finally, another important point is that the increased sensitivity for strategy-related values of managers and the decreased sensitivity for principle-related values should not be interpreted to suggest that managers are “less moral” compared to nurses. We emphasize that our conceptualization of value sensitivity favors the notion that an increased strategic sensitivity is not per se ethically less desirable. Rather, professionals of a specific occupation are expected to demonstrate a sensitivity that aligns directly with their occupation-related values. Such a sensitivity is important because a manager who is not aware of concepts including e.g. cost-effectiveness, will not succeed in his daily work. Furthermore, an insensitivity in this respect may very well induce negative consequences for employees in the institution and therefore does have ethical implications. Apart from that, managers did not differ in their sensitivity related to other, more general moral values. It would therefore be desirable to have design interventions at one’s disposal for improving professionals’ sensitivity without compromising their occupation-related thinking. In the end, such sensitivities should not substitute but supplement each other.

## Conclusions

Living a moral life is not simply a matter of following a set of learned moral rules and of learning how to apply these rules to specific situations. By emphasizing the psychological basis relevant for moral behavior, one (1) acknowledges the increased scientific understanding of the foundations of moral behavior and (2) thereby has the possibility to carve out and provide means for their specific modulation. In this research project, we attempted to incorporate current knowledge from social and moral psychology in order to develop an instrument that allows for a status-quo assessment of ones’ value sensitivity in the clinical care context that anticipates an additive possibility of specifically training the underlying competency. This idea aligns with virtue ethics by paying attention to our habits of character and developing these in order to act in a moral way.

We believe that the proposed instrument provides the following advantages: (1) previous research suggests that value-sensitivity is domain specific (see [4]). Identifying specific subcomponents of value-sensitivity for different domains (e.g. values that are especially relevant for a specific domain solely) or semantically varying values (e.g. transparency in the economic field, honesty in medicine) becomes a crucial task for developing instruments aimed at assessing and increasing value-sensitivity. (2) The instrument is thought to represent a flexibly adaptable basic module if understood as a loosely tool of interchangeable building blocks. This involves relevant values for the specific context that can be identified and enacted. The instrument can then be complemented by incorporating more — or simplified by incorporating less – values by adhering to our procedure. (3) The instrument provides a fully automated data acquisition process and could easily be extended by an automated analysis algorithm providing users with an instant status-quo assessment of ones’ moral profile. Therefore, the time consuming steps of post-coding open answers are omitted. Finally, (4) we intend to complement the instrument by an implicit measure in order to further accommodate insights gained from psychological research.

## Additional file

Additional file 1: Table S1. (a & b): Representativeness of statements in German (a) and English (b) (quality check). **Table S2** Distractor analysis: Paired sample *t*-test of mean value statements compared to distractors. **Table S3** Choices and selections by group (nurses vs professionals from management and administration) for every vignette (V1-V5) and all 11 values (first column) with respect to the aggregated number of value-selections (values) and the attributed number of points (points) to these same values. Selected Vignettes: Full text of selected vignettes used as stimulus material for the study in German and English. (PDF 587 kb)

## Footnotes

1 To exemplify calculation: If a person has (over all 5 vignettes) in the mean recognized 2 out of 4 values of the moral cluster, the “recognition rate” would be 0.5. And if this person has (again over all 5 vignettes) attributed in total 10 out of 50 possible points (i.e., 10 possible points times 5 vignettes) to those values, the normalized number of points would be 0.2. Hence, the result related to the moral values for this person would be 0.5*0.2 = 0.1.
