# Supplementary material for: Measuring value sensitivity in medicine
Source: BMC Med Ethics. 2017 Jan 28;18:5. doi: 10.1186/s12910-016-0164-7 (PMC5273831; doi:10.1186/s12910-016-0164-7)
Supplement: Additional file 1: Table S1. — (a & b): Representativeness of statements in German (a) and English (b) (quality check). Table S2 Distractor analysis: Paired sample t-test of mean value statements compared to distractors. Table S3 Choices and selections by group (nurses vs professionals from management and administration) for every vignette (V1-V5) and all 11 values (first column) with respect to the aggregated number of value-selections (values) and the attributed number of points (points) to these same values. Selected Vignettes: Full text of selected vignettes used as stimulus material for the study in German and English. (PDF 587 kb) [file 12910_2016_164_MOESM1_ESM.pdf]

Additional File 1:

Table 1a (Statements in German)

Representativeness of statements

Value	Statement	M	(SD)
Fürsorge	*Wenn eine Therapie machbar ist, soll sie auch umgesetzt werden*	*4.04*	*(1.45)*
(n = 282)	Ein Arzt oder eine Pflegekraft soll Menschen, die sich selbst nicht helfen können, Beistand leisten.	5.37	(0.88)
	Man soll sich aktiv für die Förderung von Gesundheit und Wohlbefinden von Patienten einsetzen.	5.36	(0.94)
	Ein Patient soll sich in einer Institution geborgen fühlen.	5.00	(1.09)
	Patienten sind darin zu unterstützen, wieder selbst ein gutes Leben führen zu können.	5.18	(0.97)
Leistung	Für persönliche Verdienste sollte man auch persönlich belohnt werden.	4.63	(1.21)
(n = 276)	*Auch in Abwesenheit von Regeln sollte auf einen korrekten Umgang geachtet werden.*	*4.38*	*(1.68)*
	Mitarbeiter sollen Überstunden leisten, wenn die Umstände dies erfordern.	4.29	(1.27)
	Personen sollten alles daran setzen, die gesteckten Ziele zu erreichen.	4.92	(1.13)
	Vorgesetzte sollten stets das Maximum aus ihrem Team herausholen.	4.78	(1.15)
Wirtschaftlichkeit	Institutionen im Gesundheitswesen müssen kostenbewusst arbeiten.	4.71	(1.31)
(n = 284)	Die Kosten einer Therapie dürfen beim Entscheid für oder wider ihres Einsatzes eine Rolle spielen.	4.14	(1.61)

	Ärzte sollen Aufwand und Nutzen einer medizinischen Intervention abwägen.	4.66	(1.41)
	Medizinische Mittel sollten möglichst sparsam eingesetzt werden.	3.82	(1.55)
	Die Patienten sollten einen stets korrekten Umgang erwarten können.	*3.6*	*(1.96)*
Reputation	Medizinische Institutionen sollten ihr öffentliches Image pflegen.	4.69	(1.17)
(n = 286)	Handlungen sollten dahingehend überprüft werden, wie sie von anderen beurteilt werden.	4.09	(1.50)
	Ein Arzt sollte auf seinem Gebiet eine anerkannte Kapazität sein.	4.73	(1.12)
	Eine Person ist ein zuverlässiges Mitglied seiner Gemeinschaft.	*4.02*	*(1.47)*
	Mitarbeiter sollten sich so verhalten, dass sie positiv beurteilt werden.	4.42	(1.35)
Autonomie	Ein Patient soll über eine Therapie frei von Einflüssen von aussen entscheiden.	4.95	(1.21)
(n = 282)	*Ein Arzt sollte versuchen die Ergebnisse seiner Arbeit stets zu verbessern.*	*3.85*	*(1.72)*
	Es ist anzustreben, dass Patienten ein möglichst eigenständiges Leben führen können.	5.29	(0.87)
	Auch wenn es einer Person schadet, darf diese eine medizinische Behandlung ablehnen.	5.45	(0.89)
	Der Arzt soll nicht versuchen, eigene Therapiepräferenzen dem Patienten aufzudrängen.	4.94	(1.26)
Respekt	Die Privatsphäre und Selbstbestimmung einer Person ist zu achten.	5.51	(0.74)
(n = 280)	Es sollte ein wertschätzender Umgang mit den Patienten gepflegt werden.	5.59	(0.71)
	Man sollte die Würde von Patienten achten.	5.63	(0.72)
	Patienten wie Mitarbeiter sollten hochachtungsvoll behandelt werden.	5.44	(0.79)

	Riskante Therapien sollten vermieden werden.	*2.99*	*(1.42)*
Nicht-Schaden	Andere sind vor Leid und negativen Auswirkungen zu bewahren.	5.14	(1.01)
(n = 283)	Es ist darauf zu achten, dass anderen durch eigenes Handeln nicht geschadet wird.	5.27	(1.06)
	Jegliche medizinische Intervention muss mehr Nutzen als Schaden stiften.	5.03	(1.21)
	Auch medizinische Institutionen sollten auf Rentabilität achten.	*2.92*	*(1.49)*
	Patienten sollten keinen vermeidbaren Risiken ausgesetzt werden.	5.24	(1.01)
Loyalität	Eine Person sollte zum Team und zu einer gemeinsamen Sache stehen.	5.21	(0.90)
(n = 283)	Eine Institution sollte sich auf die Treue seiner Mitarbeiter verlassen können.	4.97	(1.01)
	Auch bei schwierigen Entscheidungen unterstützt man die Entscheidung der Verantwortungsträger.	4.71	(1.07)
	Langjähriger Einsatz für eine gemeinsame Sache sollte honoriert werden.	4.82	(1.01)
	Andere sollten als selbstbestimmte und einzigartige Individuen akzeptiert werden.	*4.05*	*(1.56)*
Ehrlichkeit	Die Kommunikation mit Patienten soll aufrichtig und offen sein.	5.69	(0.61)
(n = 281)	Die Leitung einer Institution sollte seine Mitarbeiter transparent über anstehende Veränderungen informieren.	5.42	(0.83)
	Patienten und Angehörige sollten mit allen relevanten Informationen versorgt werden.	5.56	(0.78)
	Sind in einer Institution Probleme entstanden, darf die Öffentlichkeit nicht fehlinformiert werden.	5.22	(0.97)
	Eine Person soll den ihr zugewiesenen Handlungsspielraum im Griff haben.	*4.08*	*(1.61)*

Gerechtigkeit	Niemand sollte ungerechtfertigt bevorzugt werden.	5.64	(0.75)
(n = 291)	Man sollte sich anderen gegenüber so verhalten, wie man es sich auch von diesen wünschen würde.	5.17	(1.13)
	Gemeinsam definierte Regeln sind einzuhalten.	4.98	(1.13)
	Wichtige Entscheidungen sollten unvoreingenommen getroffen werden.	5.04	(1.13)
	Ein gutes Ansehen bei Patienten sollte mit allen Mitteln gewahrt werden.	*2.89*	*(1.54)*
Verantwortung	Man soll eigenständig handeln und über seine Handlungen Rechenschaft ablegen.	4.98	(1.08)
(n = 286)	Schäden, die durch die eigenen Handlungen entstehen, sollen von einem selbst übernommen werden.	4.60	(1.37)
	Die unterschiedlichen Ansprüche relevanter Gruppen sollten angemessen berücksichtigt werden.	4.19	(1.37)
	Eine medizinische Institution sollte seine Pflichten gegenüber der Gesellschaft wahrnehmen.	5.20	(0.93)
	Ein Arzt oder eine Pflegekraft soll sich um das Wohlergehen eines Patienten sorgen.	*5.16*	*(1.12)*

Remark: Distractors are indicated in *italics*.

ENGLISH TRANSLATION (translation has not been reviewed (i.e. no back-translation has been performed)):

Table 1b (statements in English)

Representativeness of statements

Value	Statement	M	(SD)
Care	*If a therapy can be realized, it should be executed.*	*4.04*	*(1.45)*
(n = 282)	A physician or a caregiver should provide assistance to patients who cannot help themselves.	5.37	(0.88)
	One should promote health and well-being of patients in a thorough way.	5.36	(0.94)
	A patient should feel save in a given institution.	5.00	(1.09)
	Patients should be supported in regaining an autonomous life.	5.18	(0.97)
Performance	Personal merits should be rewarded individually.	4.63	(1.21)
(n = 276)	*Also in the absence of explicit rules, a correct social interaction should be respected.*	*4.38*	*(1.68)*
	Coworkers should execute hours of overtime, if the circumstances require it.	4.29	(1.27)
	Individuals should do everything possible to accomplish aspired goals.	4.92	(1.13)
	Chefs should always push members of their team to the maximum/limits.	4.78	(1.15)
Cost-effectiveness	Institutions in the health care system have to work under the consideration of cost-efficiency.	4.71	(1.31)

(n = 284)	The costs of a given therapy should be of importance when evaluating decisions involving therapeutic strategies.	4.14	(1.61)
	Physicians should weigh costs and benefit for a given medical intervention.	4.66	(1.41)
	Medical goods should be used as sparingly as possible.	3.82	(1.55)
	Die Patienten sollten einen stets korrekten Umgang erwarten können.	*3.6*	*(1.96)*
Reputation	Institutions should care about their public image.	4.69	(1.17)
(n = 286)	Actions should be reviewed with regard to how others would judge those.	4.09	(1.50)
	A physician should be a known capacity in his field.	4.73	(1.12)
	A person should be a reliable member of the society.	*4.02*	*(1.47)*
	Coworkers should perform in order to be evaluated positively.	4.42	(1.35)
Autonomy	A patient should be able to give consent for a therapeutic intervention without any influences from outside.	4.95	(1.21)
(n = 282)	*A physician should try to constantly improve the results of his work.*	*3.85*	*(1.72)*
	It should be aspired that patients can live an independent life.	5.29	(0.87)
	Even if it harms an individual, he/she has the right to refuse a medical treatment.	5.45	(0.89)
	A physician should not try to force his or her medical preference on a patient.	4.94	(1.26)
Respect	The privacy and self-determination capacity of persons should be respected.	5.51	(0.74)
(n = 280)	A cherished contact with the patients should be cultivated.	5.59	(0.71)

	One should respect the human dignity of patients.	5.63	(0.72)
	Patients and coworkers should be treated respectfully.	5.44	(0.79)
	Risky therapies should be avoided.	*2.99*	*(1.42)*
Nonmaleficence	One should pay attention not to harm others.	5.14	(1.01)
(n = 283)	Others should be prevented from suffering and distress.	5.27	(1.06)
	Any medical intervention should endow more benefit than harm.	5.03	(1.21)
	Medical institutions should pay attention to aspects of profitability as well.	*2.92*	*(1.49)*
	Patients should not be exposed to avoidable risks.	5.24	(1.01)
Loyalty	A person should adhere to his team or a shared cause.	5.21	(0.90)
(n = 283)	An institution should be able to rely on its coworkers.	4.97	(1.01)
	Also in times of troubles, one supports the decisions of the people in charge.	4.71	(1.07)
	Long-term dedication/commitment for a shared cause should be rewarded.	4.82	(1.01)
	Others should be recognized as autonomous and individual / unique persons.	*4.05*	*(1.56)*
Honesty	The communication with patients or customers should be sincere and frankly.	5.69	(0.61)
(n = 281)	The leadership of an institution should inform its coworkers transparently about upcoming changes.	5.42	(0.83)
	Patients and affiliated persons should be provided with all relevant information.	5.56	(0.78)
	If there have emerged institutional problems, the public should not be misinformed.	5.22	(0.97)

	A person should master his/her assigned scope of action.	*4.08*	*(1.61)*
Justice	Nobody should be favored in an unjustified way.	5.64	(0.75)
(n = 291)	One should act according to how one would desire to be treated him/herself.	5.17	(1.13)
	Mutually defined rules should be maintained.	4.98	(1.13)
	Important decisions should be made unprejudiced.	5.04	(1.13)
	A solid reputation should be protected by all means.	*2.89*	*(1.54)*
Responsibility	One should act independently and be accountable for his actions. .	4.98	(1.08)
(n = 286)	Damage which is due to one's actions should be adopted by oneself.	4.60	(1.37)
	The diverse interests of relevant groups should be regarded appropriately.	4.19	(1.37)
	An institution should recognize its obligations towards society.	5.20	(0.93)
	A physician or a caregiver should care for the wellbeing of a patient.	*5.16*	*(1.12)*

Remark: Distractors are indicated in *italics*.

Table 2: distractor analysis

Paired sample t-test of mean value statements compared to distractors.

Value group	M_stat	M_dist	p-value
care	5.00	4.04	p = 0.000
reputation	4.09	4.02	p = 0.606
non-maleficence	5.03	2.92	p = 0.000
honesty	5.22	4.08	p = 0.000
performance*	4.63	4.38	p = 0.038
cost-effectiveness	3.82	3.60	p = 0.172
autonomy	4.94	3.85	p = 0.000
respect	5.44	2.99	p = 0.000
loyalty	4.71	4.05	p = 0.000
justice	4.98	2.89	p = 0.000
responsibility†	x	x	x

Remark: lowest mean values of value-statements (M_stat) were compared to the mean values of the distractors (M_dist) localized in the same value group. * indicates that the 2nd lowest M_stat was taken because the lowest value statement was below M_dist (see main text for explanations). For †, no comparison was performed because M_dist yielded the second highest value.

Table 3: Choices and selections by group (nurses vs professionals from management and administration) for every vignette (V1-V5) and all 11 values (first column) with respect to the aggregated number of value-selections (values) and the attributed number of points (points) to these same values. Value abbreviations: AUT=autonomy; CAR=care, CEF=cost-effectiveness, HON=honesty, JUS=justice, LOY=loyalty, NMA=nonmaleficence, PER=performance, REP=reputation, RES=respect, RPS=responsibility.

	V1				V2				V3				V4				V5			
	Nurses		Managers		Nurses		Managers		Nurses		Managers		Nurses		Managers		Nurses		Managers	
	values	points	values	points	values	points	values	points	values	points	values	points	values	points	values	points	values	points	values	points
CAR	28	25	8	4	28	38	8	6	37	91	10	12	34	40	9	9	33	66	9	5
PER	16	22	6	4	10	5	4	6	12	4	6	1	4	4	4	0	10	1	3	1
CEF	14	10	7	9	16	17	6	8	23	18	10	11	27	33	9	13	29	33	10	15
REP	20	7	6	8	15	2	7	10	16	3	7	2	17	9	5	4	12	1	7	5
AUT	15	11	3	2	30	61	10	13	35	90	10	19	33	58	8	15	31	42	9	16
RES	33	69	10	30	26	43	7	9	34	72	11	28	34	65	10	24	32	55	11	18
NMA	27	47	8	15	32	64	10	16	22	20	8	10	35	60	9	18	32	64	11	21
LOY	28	20	6	8	13	6	2	4	9	2	8	3	12	1	7	2	13	5	7	2
HON	36	99	11	19	33	79	11	18	26	37	10	10	36	89	11	14	37	70	11	21
JUS	29	40	8	6	28	33	9	15	22	24	8	7	24	7	8	11	31	27	9	4
RPS	19	20	5	5	20	22	6	5	23	9	9	5	14	4	5	0	17	6	7	2

Selected vignettes (in German):

Vignette 1:

Dr. P, ein erfahrener Onkologe in der Klinik, hat in der Vergangenheit bereits mehrfach Angebote anderer Kliniken erhalten, diese aber aufgrund der Verwurzelung an die hiesige Klinik und seiner Wohnsituation wegen, immer abgelehnt. Doch seit letzter Zeit gibt es zwischen der Klinikdirektion und ihm als Leiter des onkologischen Zentrums Unstimmigkeiten bezüglich studentischer Ausbildungsmodalitäten, patientenorientierten Informationsbestimmungen und dem Einrichten einer Palliativstation, der Dr. P. kritisch gegenüber steht. Seit dem frühzeitigen Ableben seiner Frau ist Dr. P. gegenüber den Patientinnen und Patienten noch stärker engagiert, seine Skepsis gegenüber Neuerungen ist aber ebenfalls gewachsen, was zu Konflikten mit anderen leitenden Ärztinnen und Ärzten führt. Derzeit laufen Diskussionen zu diesen Punkten zwischen Klinikdirektion und den anderen leitenden Ärzten. Unter anderem wird erwogen, ob man sich wegen der gestörten Harmonie und in Konsequenz des fehlenden einheitlichen Lösungsbestrebens besser von Dr. P. trennen sollte. Wie soll die Klinikleitung diese Situation beurteilen?

Vignette 3:

Frau J., eine Patientin mittleren Alters, muss sich infolge einer Fussgelenksverletzung einer Operation unterziehen. Obgleich eine Routineoperation, wird diese infolge Versicherungsmodalitäten von der leitenden Oberärztin der Chirurgie durchgeführt. Am Tag nach der Operation erfolgt das Austrittsgespräch, bei dem die Oberärztin und ein Krankenpfleger anwesend sind. Die Oberärztin erklärt der Patientin die Wichtigkeit einer Thromboseprophylaxe, für die das Medikament Heparin subkutan mittels einer dünnen Kanüle injiziert werden muss, ähnlich wie bei der Insulin-Selbstgabe. Vier Tage nach der Entlassung wird die Patientin notfallmässig mit Atem- und Herzrhythmusstörungen eingeliefert. Sofort wird klar, dass eine Lungenembolie vorliegt. Der Notfallarzt vermutet eine unzureichende Thromboseprophylaxe. Nachfragen bei der Patientin ergeben, dass diese nicht genau wusste, wie sie bei der Heparin-Injektion vorgehen sollte. Ein danach anschliessendes Gespräch zwischen dem Notarzt und der leitenden Oberärztin der Chirurgie ergab keine Klärung der Sachlage, da üblicherweise der Krankenpfleger die Prophylaxe erklärt, im konkreten Fall aber die Oberärztin dies offenbar tat. Die Klinikleitung muss den Fall nun begutachten und das Vorgehen der Mitarbeiter beurteilen. Auf welche Aspekte soll diese dabei achten?

Vignette 5:

Im Pflegeheim der Institution ist seit einigen Wochen ein neuer Bewohner eingetroffen, ein exzentrischer Künstler, 65 Jahre alt, der seine Atelierwohnung aufgrund zunehmender dementieller Episoden verlassen musste. Bald wird klar, dass ein gewisses Bedürfnis, seine Kunst auszuleben zu können, weiterhin besteht. Dies wiederspiegelt sich in einem ständigen Verlangen, künstlerisch tätig zu sein. Der Mann stellt die Pflegenden mit diesem Wunsch sowie seiner stark ausgeprägten Individualität zwar immer wieder vor gewisse Herausforderungen, wird ansonsten von den Pflegenden als ein auf sich bezogener, einzelgängerischen, relativ ruhigen Heimbewohner beschrieben, der ein eher zurückgezogenes Heimleben zu führen scheint. Seit kurzem ist der Mann nun kaum mehr von seiner Idee abzubringen, im Hobbyraum des Pflegeheims ein kleines Atelier

einrichten zu wollen, wo er seinem Bedürfnis, künstlerisch wirken zu können, nachgehen könnte. Dieses Bestreben, welches einiger Umstrukturierungen bedürfte, wird von einigen Mitbewohnerinnen und -bewohner unterstützt; die Mitarbeiter allerdings haben mit Blick auf Personalplanung und längerfristige Konsequenzen Bedenken. Wie soll die Leitung des Pflegeheims diese Situation beurteilen?

Vignette 6:

Prof. M. – ebenfalls Mitglied der Expertengruppe – ist eine anerkannte Forscherin im Bereich Migräne- und Schmerzforschung. Sie berichtet, sie habe kürzlich an einem Kongress von einer neuen klinischen Studie gehört, innerhalb welcher ein vielversprechender Wirkstoff getestet wird. In dieser Studie wurde auch ersichtlich, dass der Wirkstoff Verdauungsstörungen hervorrufen kann, die sich normalerweise mit einer längeren Einnahme verflüchtigen. Sie ist von den Studienerstellern direkt angesprochen und gefragt worden, ob Sie zusätzliche Patienten für die Studie rekrutieren könne. Nach der Rückkehr vom Kongress trifft sie in ihrer Sprechstunde auf Frau P., eine 45-jährige Patientin, sozial eingebunden, die seit ihrem 35. Altersjahr unter regelmässig wiederkehrenden Migräneanfällen leidet. Diese verunmöglichen es ihr zuweilen, ihren privaten wie auch beruflichen Verpflichtungen nachzukommen. Prof. M. informiert Frau P. routinemässig über das Studiendesign, worauf Frau P., von der Idee sehr angetan, sich einen Eintritt in die klinische Studie überlegt. Prof M. ist sich sicher, dass Sie aufgrund ihrer Kontakte Frau P. einen Platz in der Studie verschaffen könnte. Sie fragt die Expertengruppe, welche Informationspflichten Sie als „Schnittstelle“ zwischen Patientin und Studienteam habe. Auf was soll die Expertengruppe bei ihrer Antwort achten?

Vignette 7:

Für das zehnjährige ADHS-Kind Peter, bei dem eine Therapie mit Ritalin bislang kaum Besserung gebracht hat, gibt es die Möglichkeit eines Eintritts in eine klinische Studie, in der eine neue pharmakologische Substanz mit grossem Potential getestet werden soll. Das Studiendesign sieht vor, dass Peter regelmässig diese neue Substanz erhalten soll und im Verlauf der Studie fMRI-Scans unterzogen würde, um einfache Aufgaben im Scanner zu lösen. Dies natürlich nur auf der Grundlage, dass die medikamentöse Therapie greift, da ansonsten die 20-minütige Aufgabe im Scanner aus ärztlicher Sicht Peter nur unnötig belasten würde. Die Eltern werden mit folgenden Informationen versehen: eine Angabe über die potentiellen Nebenwirkungen der Substanz, die Freiwilligkeit des Eintritts in diese Studie, sowie die Möglichkeit, jederzeit und ohne Angabe von Gründen aus der Studie austreten zu dürfen, ohne dass ihm daraus Nachteile für die sonstige Behandlung erwachsen. Nach Einwilligung der Eltern und Beginn der Studie verbessern sich Peters Konzentrationsfähigkeiten. Im fMRI-Scan stellen die Ärzte jedoch dann eine Anomalie im Bereich des Frontalhirns fest, hervorgerufen allenfalls durch eine frühkindliche Hirnblutung. Nun wird diskutiert, ob Peter besser von der Studie ausgeschlossen werden soll, weil die Aussagekraft der Studie beeinträchtigt werden könnte. Auf welche Aspekte soll die Expertengruppe bei der Untersuchung dieser Sachlage achten?

Selected Vignettes (in English; translation has not been reviewed (i.e. no back-translation has been performed)):

Vignette 1:

Dr. P, an experienced oncologist in hospital X has previously received several offers from various other hospitals. So far, he always declined these offers because of his relatedness to his hospital and due to his housing situation. However, for some time, internal disagreements between him, as a director of the oncology unit, and the clinical director, emerged. They concern student-based education modalities, patient-oriented information requirements and the set-up of a palliative care unit towards which Dr. P. has a critical stance. Since the untimely death of his wife, Dr. P. is even more engaged towards the patients in his unit. Simultaneously, his skepticism towards innovation increased. This resulted in several conflicts with other senior physicians. Negotiations with the executive board of the hospital and the other senior physicians regarding the mentioned issues are currently underway. Among other things, the clinic director is considering whether Dr. P. should be dismissed due to the disturbed harmony within the hospital and the lack of consensual procedures. How should the clinic management evaluate this situation?

Vignette 3:

Miss J., a middle-aged patient has to undergo surgery following ankle injury. Although being a routine operation, the senior surgeon is performing the surgery because of insurance modalities. The day after surgery, the exit interview takes place in the presence of the senior surgeon and the nurse. The senior physician explains the importance of thrombosis prophylaxis and the procedure of subcutaneous heparin-injection by means of a thin needle, similar to insulin self-application. Four days after having left the hospital, the patient is transferred to the hospital for emergency medical treatment due to respiratory dysfunction and cardiac arrhythmia. Immediately, a pulmonary emoblism is diagnosed. The emergency doctor assumes an insufficient thrombosis prophylaxis being the cause for the lung embolism. Subsequent enquiries revealed that the patient apparently was insecure regarding the procedure of heparin-injection. A subsequent discussion with the senior physician revealed no clarification of the situation. Usually the nurse explains the thrombosis prophylaxis. In the current situation however, the senior physician apparently has taken over this task. The clinic management now has to examine the case and has to evaluate the course of action of the personnel. On which aspects should they focus?

Vignette 5:

A 65 years old eccentric artist has been admitted to a nursing home as a consequence of increasingly frequent episodes of dementia. It quickly becomes clear that there is still a need for him to express himself through his passion for art, particularly evidenced by a permanent mentioning of his desire. Even though the handling of the artist often was challenging for the nurses due to his distinctive personality and his desire for artwork, he otherwise is described as a solitary, introverted and relatively calm home resident. Recently, he pushes forward the idea of establishing a studio in the hobby room of the nursing home in order to live out his desire for artwork. Some of the other residents support this idea. However, as the construction of a studio would imply several

restructuring work and due to staff planning and potential long-term consequences, the personnel is hesitant regarding this idea. How should the management of the nursing home evaluate this situation?

Vignette 6:

Professor M., a member of the expert committee, is a renowned scientist in the field of migraine and pain research. She reports that she recently attended a congress, where a new clinical study testing a promising drug for migraine has been presented. In this presentation of the study, it was mentioned that the drug might induce side-effects including digestive dysfunctions. However, these normally disappear during long-term use. Prof. M. has been asked by the study director, whether she would be able to recruit patients for the study. After the return of the congress, a 45 years old patient was reported to Prof. M. for consultation. Miss P., the patient, is a socially engaged person suffering since the age of 35 from recurrent, regularly appearing migraine. Miss P. is often unable to work and to take care of her personal duties due to the re-emerging migraines. Prof. M. informs the patient routinely about the study design. Showing great interest in the study, the patient considers entering it. Prof. M. is confident to be able to secure a spot in the study because of her contacts to the study director. She now asks the expert committee regarding her information requirements between patient and study-team. On which aspects should the expert committee pay attention?

Vignette 7:

Peter, a 10 years old patient suffering from ADHD for whom a therapy including Ritalin has largely failed, would have the possibility to enter a clinical study testing a new pharmacological compound with great potential. The study design envisages a regular intake of this new substance combined with fMRI-scans during which Peter is supposed to solve a simple task. The 20-minutes fMRI task is only permissible from a medical point of view, if the pharmacological therapy shows beneficial effects; otherwise, the procedure would be an unnecessary burden for Peter. The parents are provided with the following information: details on possible side-effects of the drug, the voluntariness of study entrance and the possibility to quit the study any time without the need of stating reasons and with no disadvantages with respect to other medical treatments. After parental approval the concentration abilities of Peter improve. In the fMRI-scan the physicians however detect a malformation situated in the region of the frontal brain, possibly caused by an early childhood cerebral hemorrhage. Now the debate concerns whether Peter should better be excluded from the study because of impairment of the study's conclusiveness (significance/validity of the study). On which aspects should the expert committee pay attention when investigating this situation?
